# A prospective, single-center, quasi-experimental study protocol for evaluating the efficacy of stepwise Yalom group therapy in reducing depressive symptoms and interpersonal problems in Chinese female patients with depressive disorders

**DOI:** 10.3389/fpsyg.2026.1831835

**Published:** 2026-05-07

**Authors:** Yue Zhou, Bingyi Du, Jixuan Hou, Runing Hou, Hongyan Yang, Chang Guo, Yangyang Yu, Yanping Zhang, Suhong Wang, Huimin Zhang, Fang Yan, Chuansheng Wang

**Affiliations:** 1 The Second Affiliated Hospital of Henan Medical University (Henan Mental Hospital), Xinxiang, Henan, China; 2 School of Nursing, Henan Medical University, Xinxiang, Henan, China; 3 Henan Collaborative Innovation Centre of Prevention and Treatment of Mental Disorder, Xinxiang, Henan, China

**Keywords:** depressive disorder, female, interpersonal difficulties, protocol, quasi-experimental studies, Yalom group therapy

## Abstract

**Background:**

Depressive disorder is affecting significantly more women than men. It is often accompanied by interpersonal difficulties, which severely impair patients’ social functioning. Individual interpersonal therapy can help improve interpersonal problems, but the implementation of individual therapy is more difficult than that of group therapy. This study aims to evaluate the efficacy of Yalom group therapy in reducing depressive symptoms and interpersonal difficulties among Chinese women with depressive disorders.

**Methods:**

A total of 90 participants will be allocated to either the intervention group or the control group. Inclusion criteria: ① Diagnosis of depressive disorder or recurrent depressive disorder according to the International Classification of Diseases, 10th Revision (ICD-10), confirmed by two attending physicians or above; ② Female sex; ③ Age ≥18 years and <60 years; ④ Ability to communicate verbally; ⑤ Hamilton Depression Scale-17 (HAMD-17) score ≥18; ⑥ No psychological treatment received within 6 months; ⑦ Voluntary participation with informed consent. Exclusion criteria: ① Current or planned physical therapy (e.g., electroconvulsive therapy, deep transcranial magnetic stimulation); ② Participation in other group or individual psychological therapy; ③ Comorbidity with severe conditions (e.g., severe personality disorders, substance abuse, neurological diseases); ④ Intellectual disability. The control group will receive 5 weeks of routine care and standard treatment. The intervention group will receive 5 weeks of stepwise Yalom group therapy plus routine care and standard treatment. Primary outcomes include improvements in depressive symptoms and interpersonal difficulties. Secondary outcomes include reductions in anxiety symptoms and enhancements in social functioning. Statistical analyses will be performed using SPSS 27.0. Data will be analyzed in accordance with statistical principles, and missing data will be handled using multiple imputation. *p* < 0.05 will be considered statistically significant.

**Discussion:**

This study will enhance understanding of the application of Yalom group therapy in female patients with depressive disorders. As the first study in China to implement a full-course intervention for female inpatients with depressive disorders, it will provide insights into the utility of Yalom group therapy for mitigating disease severity and improving interpersonal outcomes in this population.

**Clinical trial registration:**

https://www.chictr.org.cn/, identifier ChiCTR2600116067

## Highlights

**What is already known**

- Yalom group therapy has been shown to alleviate depressive and anxiety symptoms across various clinical populations.
- Interpersonal difficulties are highly prevalent among female patients with depressive disorder and contribute to poor social functioning.

**What this paper adds**

- This is the first study to implement a full-course stepwise Yalom group therapy protocol tailored specifically for female inpatients with depressive disorder in China.
- It introduces a structured two-phase intervention (low-functioning followed by high-functioning group therapy) designed to address both disease severity and interpersonal functioning during hospitalization.

## 1 Introduction

Depressive Disorder is a mental condition characterized by persistent low mood, diminished interest or pleasure, and a recurrence rate as high as 75 ~ 90%, rendering it a lifelong illness (Monroe and Harkness, 2022). It causes substantial impairment in cognitive and social functioning, adversely affecting patients’ quality of life, work, study, and family relationships (Fisenko et al., 2025). Women are disproportionately affected: the number of female patients with depressive disorder surged from 49.6 million in 1990 to 85.6 million in 2021 (Liao et al., 2025), with a projected age-standardized incidence rate of 2465.8 per 100,000 by 2030 (Shen et al., 2025).

This gender disparity is closely linked to women’s unique biological, psychological, and social characteristics. Biologically, reproductive traits such as age at menarche, first sexual intercourse, and first childbirth are significant risk factors (Wang et al., 2023); Socially, within the Chinese cultural context, women’s multifaceted roles in family and society increase their vulnerability to depressive disorder (Ling et al., 2025). Thus, targeted intervention research for female patients with depressive disorder holds important public health significance.

In addition to core symptoms such as low mood, hopelessness, and suicidal ideation (O'Mahony et al., 2024), patients with depressive disorder frequently suffer from interpersonal difficulties (Liao et al., 2025; Shen et al., 2025). Studies have shown that 62.4% of female patients with depressive disorder experience varying degrees of interpersonal problems (Köktaş and Taşdemir, 2025). These difficulties are both a consequence and a potential cause of depression, forming a vicious cycle (Xu et al., 2025). Improving interpersonal functioning has been proven to alleviate depressive symptoms and promote the recovery of social functioning (Cuijpers et al., 2021), making it a key component of comprehensive treatment for female patients with depressive disorder.

Interpersonal Psychotherapy (IPT) is a well-established intervention for interpersonal difficulties in depression, focusing on resolving current interpersonal issues through individual therapy. Chinese scholars such as Luo et al. (2025) have developed expert consensus on IPT for depressive disorder in China. However, due to shortages of mental health professionals (Kang et al., 2024) and uneven distribution of rehabilitation resources (Lian et al., 2025) in China, one-on-one individual therapy cannot meet the large-scale needs of patients with depressive disorder. In this context, group therapy emerges as a cost-effective alternative.

Irvin Yalom posited that, Irvin (2015a) “all human distress stems from interpersonal conflicts, and the optimal resolution lies in leveraging group dynamics to address them”. His group therapy model creates a safe, supportive “interpersonal laboratory,” where therapists act as transparent participants rather than authoritative leaders. This allows members to observe, experience, and modify maladaptive interaction patterns through “here-and-now” authentic interactions and feedback. By countering feelings of isolation (“I am alone in my suffering”), group members gain social confidence and skills through curative factors such as universality, hope, and altruism (Irvin, 2015a; Qiu, 2017). This relationship-based learning process is difficult to replicate in individual therapy (Irvin, 2015b). Yalom categorized inpatient groups into low-functioning and high-functioning levels, outlining specific protocols for each (Irvin, 2015a).

Yalom group therapy has demonstrated efficacy in diverse populations, including patients with depressive disorder, anxiety disorder, and cancer. For example, Li et al. (2018) applied Yalom’s interpersonal dynamic group model to patients undergoing tongue cancer surgery, effectively reducing anxiety and depression. Zhao et al. (2023) conducted a brief focused group therapy study (aligned with Yalom’s emphasis on interpersonal learning and group dynamics) and reported positive outcomes for interpersonal functioning and depressive symptoms in patients with depression. Yalom group therapy has also been widely used in non-clinical settings for psychological growth and supportive intervention—for instance, Jiang (2024) integrated Yalom group counseling into a university college system, finding improvements in college students’ interpersonal awareness and self-identity.

Given its proven effectiveness in alleviating depression, anxiety, and interpersonal difficulties across various populations, stepwise Yalom group therapy is expected to yield favorable outcomes for female patients with depressive disorder.

To date, no studies in China have been implemented a full-course psychological intervention for female inpatients with depressive disorder. This study aims to fill this gap by delivering a comprehensive intervention during hospitalization and evaluating improvements in disease severity and interpersonal functioning. The intervention is based on Irvin Yalom’s existential psychotherapy theory (Qiu, 2017) and his protocols for low- and high-functioning group therapy (Irvin, 2015a), refined through expert panel discussions. The control group will receive routine care.

This study seeks to provide empirical evidence for optimizing psychological treatment protocols for female inpatients with depressive disorder in China. Specific objectives include:

(1) Developing a “stepwise Yalom group therapy” protocol;
(2) Evaluating the protocol’s efficacy in reducing disease severity and improving interpersonal difficulties among female patients with depressive disorder.

## 2 Methods

### 2.1 Study design

**Figure 1:**
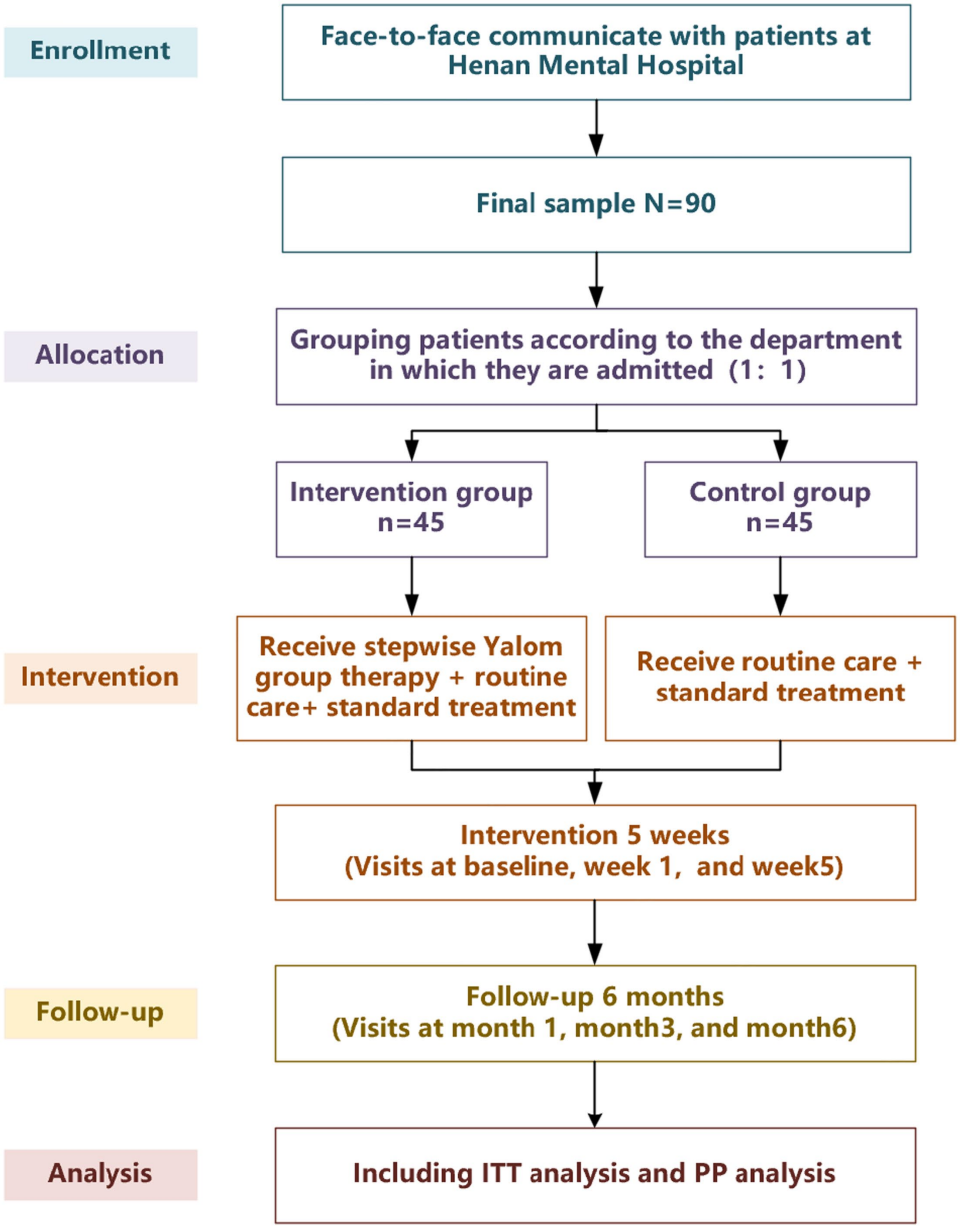
Flow diagram.

This is a quasi-experimental study conducted at The Second Affiliated Hospital of Henan Medical University, using quantitative methods. Outcome measures include the Hamilton Depression Scale-17 (HAMD-17), Beck Depression Inventory (BDI), Hamilton Anxiety Scale-14 (HAMA-14), Inventory of Interpersonal Problems-32 (IIP-32), and Scale of Social Function in Psychosis Inpatients (SSPI). The study is scheduled to run from January 2026 to December 2026, consisting of a 5-week intervention phase followed by a 6-month follow-up period. Data collection will occur at baseline (pre-intervention, t0), mid-intervention (Week 1, t_1_), post-intervention (Week 5, t_2_), and 1, 3, and 6 months (t_3_, t_4_, t_5_) after intervention completion. The control group will receive routine care and standard treatment, while the intervention group will receive routine care, standard treatment, and stepwise Yalom group therapy. The study flow diagram is shown in Table 1 and Figure 1.

**Table 1:** Timeline for study enrollment, intervention, and evaluation.

Item	Study period							
	Enrollment	Allocation	Post-allocation			Close-out		
Timepoint	−t	0	t_0_	t_1_	t_2_	t_3_	t_4_	t_5_
Enrolment								
Informed consent	×							
Eligibility screen	×							
Allocation		×						
Interventions								
Control group			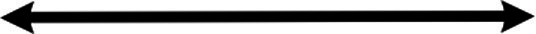					
Intervention group			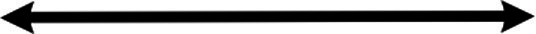					
Assessments								
HAMD-17			×	×	×			
BDI			×	×	×	×	×	×
HAMA-14			×	×	×			
IIP-32			×	×	×	×	×	×
SSPI			×	×	×			

HAMD-17, Hamilton Depression Scale-17; BDI, Beck Depression Inventory; HAMA-14, Hamilton Anxiety Scale-14; IIP-32, Inventory of Interpersonal Problems-32; SSPI, Scale of Social Function in Psychosis Inpatients.

### 2.2 Study setting, randomization, and blinding

This is a single-center, single-blind, quasi-experimental study using cluster randomization by ward. The rationale for ward-based allocation is as follows: ① Prevention of contamination. Patients within the same ward have frequent daily contact; if some patients received group therapy while others did not, they could share intervention content, diluting the true treatment effect. ② Group dynamics of Yalom therapy. Yalom group therapy relies on building cohesion within a natural inpatient unit; mixing intervention and control participants in the same ward would undermine group processes and introduce resentment. ③ Clinical feasibility. Ward routines, nursing schedules, and rehabilitation activities are organized at the ward level; assigning two different treatment protocols within one ward would disrupt clinical operations. Therefore, five wards (the hospital conducting this research only had 5 female patient wards) were randomly assigned using Excel (SORTBY, ROW, RANDARRAY functions) to either the intervention group (1 ward), control group (1 ward), or excluded (3 wards). This cluster randomization reduces contamination risk while preserving the ability to compare outcomes between groups. Baseline comparability between the two selected wards will be tested, and any remaining imbalance will be adjusted using ward-level covariates in the statistical model.

A single-blind design will be adopted: only the participants will be unaware of their group allocation, while assessors will be informed. All participants’ activities will be conducted under the heading of “participating in ward activities.”

### 2.3 Study participants

The study has been approved by the Ethics Committee of The Second Affiliated Hospital of Henan Medical University. Participants will be recruited from The Second Affiliated Hospital of Henan Medical University starting in January 2026. After face-to-face communication, eligible patients will sign an informed consent form to participate. Recruitment and intervention will occur in waves, with all participants completing the 5-week intervention.

#### 2.3.1 Inclusion criteria

(1) Diagnosis of depressive disorder or recurrent depressive disorder according to International Classification of Diseases 10th Revision (ICD-10), confirmed by two attending physicians or above;
(2) Female sex;
(3) Age ≥18 years and <60 years (Wang et al., 2023);
(4) Ability to communicate verbally;
(5) HAMD-17 score ≥18;
(6) No psychological treatment received within 6 months (Wang et al., 2023);
(7) Voluntary participation with informed consent.

#### 2.3.2 Exclusion criteria

(1) Current or planned physical therapy (e.g., electroconvulsive therapy, deep transcranial magnetic stimulation);
(2) Participation in other group or individual psychological therapy;
(3) Comorbidity with severe conditions (e.g., severe personality disorders, substance abuse, neurological diseases);
(4) Intellectual disability.

### 2.4 Sample size

Sample size was calculated using a two-sample *t*-test formula $n=2\times {\left(\frac{(z_{1}-\alpha /2+z_{1}-\beta )\times \sigma }{\delta }\right)}^{2}$ , referencing results from a group IPT intervention study in patients wit h depressive disorder (Wang et al., 2023). Setting *α* = 0.05, *β* = 0.2, Z₁₋α/₂ = 1.96, Z₁₋β = 0.84, *δ* = 3.8 (mean difference between the control and intervention groups: 10.74–6.94), and *σ*= $\sqrt{\frac{(n_{1}-1)s_{1}^{2}+(n_{2}-1)s_{2}^{2}}{n_{1}+n_{2}-2}}$ ≈4.86 (*n*_1_ = 52, *S*_1_ = 4.59, *n*_2_ = 49, *S*_2_ = 5.14), the required sample size per group was approximately 36. Accounting for a 20% attrition rate, 45 participants per group (90 total) will be recruited.

### 2.5 Intervention

#### 2.5.1 Intervention team

The team consists of 1 principal investigator, 3 psychiatrists, 2 mental health rehabilitation therapists, 3 psychiatric nurses, and 2 research assistants. The principal investigator oversees study design and implementation; psychiatrists are responsible for diagnosis and pharmacotherapy; rehabilitation therapists deliver the intervention; psychiatric nurses monitor the quality of routine care; research assistants conduct literature reviews, take meeting minutes, document intervention processes, and collect data at specified time points.

#### 2.5.2 Control group intervention

The control group receives routine care and standard treatment. Routine care includes daily living support, dietary and sleep management, safety monitoring, and daily rounds by responsible nurses to assess psychological status and behavioral changes. Standard treatment involves daily rounds by physicians, who prescribe medications approved by China’s National Medical Products Administration for depressive disorder (e.g., paroxetine, sertraline, fluoxetine), and the specific choice of medication and dosage shall be based on the doctor’s recommendation.

#### 2.5.3 Intervention group intervention

The intervention group receives routine care, standard treatment, and stepwise Yalom group therapy—comprising 1 week of low-functioning group therapy followed by 4 weeks of high-functioning group therapy (Table 2). The intervention content for the intervention group and control group is shown in Table 3. A more detailed treatment structure is provided in the Supplementary materials.

**Table 2:** Core components of stepwise Yalom group therapy.

Phase	Duration and frequency	Key components	Objectives
Low-functioning group therapy (Week 1)	5 sessions/week, 30 ~ 35 min/session	Self-disclosure	Encourage members to share openly in a safe environment, fostering interaction and mutual understanding
		Empathy	Cultivate awareness of others’ feelings, enhancing group cohesion
		Here-and-now interaction	Promote awareness of group dynamics, facilitating immediate feedback and understanding
		Didactic discussion	Summarize simple emotion regulation strategies to improve self-care abilities
		Self-change	Encourage reflection on personal growth and development of change plans with group support
High-functioning group therapy (Weeks 2–5)	3 sessions/week, 60–75 min/session	Exploration of personal issues	Help patients identify and modify maladaptive interpersonal patterns, learn new social skills through here-and-now interactions, and alleviate interpersonal distress

**Table 3:** Intervention comparison between groups.

Time	Control group	Intervention group
Week 1	Routine care + Standard treatment	Routine care + Standard treatment + Low-functioning group therapy
Week 2	Routine care + Standard treatment	Routine care + Standard treatment + High-functioning group therapy
Week 3	Routine care + Standard treatment	Routine care + Standard treatment + High-functioning group therapy
Week 4	Routine care + Standard treatment	Routine care + Standard treatment + High-functioning group therapy
Week 5	Routine care + Standard treatment	Routine care + Standard treatment + High-functioning group therapy

It should be noted that the differences in treatment frequency and duration between the low-functioning stage (Week 1: 5 times per week, 30–35 min each) and the high-functioning stage (Week 2–5: 3 times per week, 60–75 min each) were deliberately designed and based on clinical evidence. The design basis mainly includes three points: ① Patients in the low-functioning stage (Week 1) usually have acute depression of moderate to severe severity, showing symptoms such as psychomotor retardation, fatigue, poor concentration, and reduced verbal initiative. A shorter single treatment duration (30–35 min) can avoid patients experiencing mental fatigue and frustration, while a higher treatment frequency (performed daily) can provide a stable treatment structure and create opportunities for isolated and withdrawn patients to repeatedly engage in basic social contact, which has therapeutic significance. ② When entering the high-functioning stage (Week 2–5), patients have partially relieved symptoms after drug treatment and routine ward care, and can sustain attention for 60–75 min. They can participate in deeper interpersonal exploration, role-playing, and feedback training. A lower treatment frequency (3 times per week) allows patients to leave intervals for reflection, practicing new interpersonal skills in the ward, and consolidating comprehension content. This design conforms to the principle of interval learning in psychological treatment. ③ Although the time allocation methods are different, the total weekly treatment duration of both stages is similar. This phased dosage design follows the relevant suggestions proposed by Yaron for inpatient group therapy and has been verified in our pre-test (see Table 4). In the test, patients tolerated both treatment forms well and showed clinical symptom improvement.

**Table 4:** Pilot study results.

Patient ID	Age	Time point	HAMD-17	BDI	HAMA	IIP-32	SSPI
1	26	Pre-intervention	26	5	18	18	34
		Post-intervention	20	1	14	7	41
2	28	Pre-intervention	19	9	15	28	32
		Post-intervention	16	9	12	25	33

#### 2.5.4 Modification of interventions

Participants retain the right to droupout voluntarily at any time. Intervention for individual patients will be suspended or modified if:

(1) The patient droupout voluntarily;
(2) Severe adverse events occur during treatment;
(3) Researchers determine that discontinuing treatment is in the patient’s best interest (e.g., excessive burden from participation).

Decisions to withdraw or modify interventions will be made collectively by the research team. All withdrawals and modifications will be documented, including reasons, communication details, and follow-up actions, in compliance with ethical requirements for privacy protection.

#### 2.5.5 Operationalization of low-functioning and high-functioning phases

In this study, the term low-functioning group therapy refers to the initial inpatient phase, during which participants typically present with relatively severe depressive symptoms, reduced psychological energy, limited concentration, and reduced tolerance for emotionally intense interpersonal exploration. Therefore, sessions in Week 1 are shorter, more structured, and more supportive, with emphasis on safety, engagement, basic emotional expression, empathic listening, and simple here-and-now interaction.

High-functioning group therapy refers to the subsequent phase in which participants have achieved preliminary clinical stabilization and are able to maintain attention for longer sessions, participate in reciprocal interaction, reflect on interpersonal patterns, and tolerate feedback from other group members. Therefore, sessions in Weeks 2–5 are longer and place greater emphasis on interpersonal learning, here-and-now process exploration, feedback exchange, and modification of maladaptive relational patterns.

Progression from the low-functioning phase to the high-functioning phase was based on predefined clinical readiness criteria, including the ability to attend sessions consistently, communicate coherently, tolerate group interaction without marked behavioral dysregulation, and engage in basic self-reflection and interpersonal feedback.

### 2.6 Measures to reduce attrition

To promote participation and reduce attrition:

(1) During hospitalization, nurses will verbally remind patients of upcoming sessions and escort them to interventions;
(2) After discharge, research assistants will send reminders for follow-up via phone, text message, or email;
(3) Regular post-discharge contact will be maintained to address patient concerns and ensure ongoing participation;
(4) Small incentives (e.g., snacks, daily necessities) will be provided to encourage retention and timely follow-up.

If the respondents intend to droupout, then collect the last outcome indicators before their withdrawal and the reasons for their withdrawal.

### 2.7 Development of the stepwise Yalom group therapy protocol

#### 2.7.1 Draft protocol development

The research team developed an initial draft based on Irvin Yalom’s existential psychotherapy theory (Qiu, 2017), his protocols for low- and high-functioning group therapy (Irvin, 2015a), and clinical realities (e.g., average Level 1 care duration of 119 h and hospital stay of 33 ~ 37 days for inpatients with depressive disorder at the study site).

#### 2.7.2 Revised protocol development

To enhance scientific rigor, feasibility, and clinical applicability, a panel of 15 experts (with expertise in mental health rehabilitation, psychiatric nursing, and psychiatry/mental health; all holding intermediate or senior titles and ≥10 years of experience, Table 5) was convened to review and revise the draft. Experts received the draft and supporting materials 2 days prior to the meeting, during which the research team presented the design rationale, and experts provided feedback followed by group discussion. The draft was revised based on expert recommendations to form a revised version.

**Table 5:** Demographic characteristics of expert panel members.

Characteristic	Category	*n* (%)
Age (years)	35 ~ 39	1 (6.67%)
	40 ~ 44	4 (26.67%)
	45 ~ 49	4 (26.67%)
	50 ~ 54	1 (6.67%)
	55~	5 (33.33%)
Sex	Male	2 (13.33%)
	Female	13 (86.67%)
Education	College diploma	2 (13.33%)
	Bachelor’s degree	10 (66.67%)
	Master’s degree	2 (13.33%)
	Doctoral degree	1 (6.67%)
Field of expertise	Mental health rehabilitation	6 (40.00%)
	Psychiatric nursing	7 (46.67%)
	Psychiatry/mental health	2 (13.33%)
Work experience (years)	10 ~ 14	2 (13.33%)
	15 ~ 19	0 (0.00%)
	20 ~ 24	4 (26.67%)
	25 ~ 29	1 (6.67%)
	30 ~ 34	5 (33.33%)
	35~	3 (20.00%)
Professional title	Intermediate	1 (6.67%)
	Associate senior	9 (60.00%)
	Senior	5 (33.33%)

#### 2.7.3 Final protocol development

A pilot study was conducted to verify feasibility and safety. Two female inpatients with depressive disorder (meeting inclusion/exclusion criteria) completed 1 week of low-functioning and 1 week of high-functioning group therapy after signing informed consent. Rehabilitation therapists and researchers documented interactions and adverse events after each session. Pre- and post-pilot data indicated positive effects on disease severity, interpersonal difficulties, and social functioning (Table 4), leading to the final protocol.

### 2.8 Outcome measures

During the patient’s hospital stay, questionnaire data were collected in person. Following discharge, researchers would relay questions to participants and record their responses. Demographic information was gathered using a self-designed questionnaire. The primary outcome measure was disease severity, assessed via the HAMD-17, the BDI, and the HAMA-14. Secondary outcome measures comprised interpersonal difficulties and social functioning, evaluated, respectively, using the IIP-32 and the SSPI.

(1) Demographic Questionnaire

Developed by the research team, this questionnaire collects information including gender, age, height, weight, ethnicity, religious beliefs, marital status, education level, occupation, only-child status, medical history, and number of hospitalizations. To ascertain the patient’s general condition.

(2) Hamilton Depression Scale-17 (HAMD-17)

Developed by Hamilton (1960), this widely used scale assesses depressive severity in adults. It comprises 5 factor domains: ① Anxiety/somatization, ② Weight loss, ③ Cognitive impairment, ④ Retardation, ⑤ Sleep disturbance. Most items use a 5-point Likert scale (0 = “none” to 4 = “severe”), while a few use a 3-point scale (0 = “none” to 2 = “severe”). Total scores range from 0 to 52: 8 ~ 17 (mild depression), 18 ~ 24 (moderate depression), ≥25 (severe depression) (Li et al., 2026). The scale has high reliability (Cronbach’s *α* = 0.88 ~ 0.99, *p* < 0.01) and criterion validity (0.92).

(3) Beck Depression Inventory (BDI)

Developed by A. T. Beck and adapted into Chinese by Hongbo Zheng et al. (Zheng and Zheng, 1987), this 13-item self-report scale assesses depressive severity. Items use a 4-point scale (0 = “no symptom” to 3 = “severe”). Total scores: 0 ~ 4 (minimal), 5 ~ 7 (mild), 8 ~ 15 (moderate), ≥16 (severe). Cronbach’s *α* = 0.846.

(4) Hamilton Anxiety Scale-14 (HAMA)

Developed by Hamilton (1960), this clinician-rated scale assesses anxiety severity. Items use a 5-point Likert scale (0 = “no symptom” to 4 = “extreme”). Total scores: <7 (no anxiety), 7–14 (possible anxiety), 14 ~ 21 (definite anxiety), 21 ~ 29 (marked anxiety), >29 (severe anxiety). The 14-item version has a cutoff score of 14. Cronbach’s α = 0.820.

(5) Inventory of Interpersonal Problems-32 (IIP-32)

Developed by Soldz et al. and adapted into Chinese by Li (2009), this scale assesses current interpersonal difficulties across 8 domains: Dominance /Control (PA), Competition /Self-centeredness (BC), Detachment /Distance (DE), Social Avoidance /Isolation (FG), Submissiveness /Inassertiveness (HI), Overaccommodation/Exploitability (JK), Self-sacrifice /Overcare (LM), Intrusiveness /Overexpression (NO). The 32 items use a 5-point scale (0 = “not at all” to 4 = “extremely”). Higher scores indicate greater interpersonal difficulties. Test–retest reliability ranges from 0.61 to 0.83, and Cronbach’s *α* = 0.89.

(6) Scale of Social Function in Psychosis Inpatients (SSPI)

Developed by Zhou et al. (2004) to assess social functioning in inpatients, this 12-item scale comprises 3 factors: ① Daily living skills (Items 1 ~ 3), ② Activity and social interaction (Items 4 ~ 8), ③ Social activity skills (Items 9 ~ 12). Items use a 5-point scale (0 = “function absent” to 4 = “excellent function”). Higher scores indicate better social functioning. Factor I and III are rated based on 1-week performance; Factor II is rated over 1 month due to variability. Social function impairment grades: <18 (severe), 18 ~ 28 (moderate), 29 ~ 38 (mild), >38 (normal). Inter-rater reliability (Kappa = 0.79 ~ 0.92) and test–retest reliability (0.722) are good.

### 2.9 Statistical analysis

Data will be analyzed using SPSS 27.0. Categorical variables will be described as frequencies (percentages), and continuous variables as mean ± standard deviation (normal distribution) or median (interquartile range) (non-normal distribution). Between-group comparisons of baseline continuous data will use independent samples t-tests (normal distribution, equal variance) or Mann–Whitney U tests (otherwise). Categorical data will be compared using chi-square tests or Fisher’s exact tests (if >20% of cells have expected frequencies <5).

Repeated-measures analysis of variance (ANOVA) will be used to compare scale scores between groups pre- and post-intervention, with “time” as the within-subjects factor and “group” as the between-subjects factor. Mauchly’s test of sphericity will be performed; if violated, the Greenhouse–Geisser correction will be applied. The primary focus will be the “time × group” interaction; if significant, simple effects analysis will be conducted. Generalized estimating equations will be used as a robustness check if ANOVA assumptions are severely violated.

Intention-to-treat (ITT) analysis will include all randomized participants regardless of intervention completion. Multiple imputation methods shall be employed to supplement missing data. Per-protocol (PP) analysis will include only participants who completed the intervention and follow-up. All tests are two-tailed, with *p* < 0.05 considered statistically significant.

### 2.10 Study management

#### 2.10.1 Data management

All study outcomes will be documented in case report forms (CRFs) to ensure accuracy and traceability. Data will be collected by trained researchers in accordance with the ethics-approved protocol. Participant information will be strictly confidential.

After study completion, data and related materials will be archived long-term in compliance with relevant laws and regulations. Data will be encrypted to prevent unauthorized access. All personnel involved in data management will receive training and sign confidentiality agreements. The principal investigator and data manager will jointly oversee data entry and storage.

During analysis, data will be cleaned and preprocessed. Missing data will be handled using appropriate statistical methods. All analyses will be performed using software in accordance with a pre-specified analysis plan.

#### 2.10.2 Adverse event collection and assessment

Adverse events include negative reactions, psychological distress, social impacts, or other unforeseen consequences related to the intervention. The research team will monitor and collect adverse event data throughout the study. Participants will be informed to report any adverse reactions confidentially at any time.

Data will be collected via face-to-face interviews, questionnaires, and phone calls to ensure comprehensive identification of potential harms. Clinical teams will assess whether adverse events are intervention-related and classify their severity (Grade 1 ~ 4):

Grade 1 (Critical event): Unexpected death or permanent functional loss not due to natural disease progression;
Grade 2 (Adverse outcome): Physical or functional harm caused by treatment rather than the disease;
Grade 3 (No-harm event): Error occurred but caused no harm or only minor, self-resolving symptoms;
Grade 4 (Near-miss event): Error identified and corrected before causing harm.

Grade 1 or 2 adverse events will be reported immediately to the Ethics Committee for review. Appropriate actions will be taken in consultation with the committee and study clinicians. Including but not limited to measures such as negotiating compensation with the subjects.

#### 2.10.3 Ethics and regulatory compliance

The study will be conducted in accordance with local laws and regulations and has been approved by the Ethics Committee of The Second Affiliated Hospital of Henan Medical University.

All researchers and staff will receive training on the protocol, modifications, interventions, and their responsibilities. A list of co-investigators and qualified personnel authorized to perform key tasks will be maintained.

### 2.11 Data monitoring

A formal Data Monitoring Committee (DMC) will not be implemented for this study. This decision is justified by the low inherent risk profile of the research intervention, the simple, non-invasive study design, and the absence of planned interim efficacy or safety analyses that would trigger independent data oversight. The study involves minimal risk to participants, with no anticipated serious adverse events associated with the intervention, and all safety monitoring will be performed in real time by the principal investigators and the Ethics Committee of the Second Affiliated Hospital of Henan Medical University through routine periodic safety reports and protocol compliance reviews.

### 2.12 Fidelity monitoring

To enhance intervention fidelity, a study-specific fidelity checklist was developed based on the intervention manual and published treatment fidelity recommendations (Bellg et al., 2004; Perepletchikova, 2011; Lopez-Alcalde et al., 2022). The checklist was used to assess adherence to session structure, core therapeutic tasks, therapist behaviors, and phase-specific intervention principles. All group therapy sessions will be audio-recorded. Senior therapists will randomly select recordings for fidelity evaluation every 2 weeks. If the score for a single session reaches 80% or more of the total score, it is considered as meeting the compliance criteria. Therapists will receive weekly supervision based on the compliance feedback; if the compliance rate for two consecutive sessions is below 80%, remedial training will be provided for them.

### 2.13 Quality control

All researchers will complete unified training on the study protocol, core tenets of Yalom’s existential psychotherapy, scale administration, intervention procedures, and emergency management. Only those who pass post-training assessments will participate. Rehabilitation therapists delivering the intervention will have received specialized training in low- and high-functioning group therapy, with additional homogenization training prior to the study to ensure consistency and safety.

Intervention venues and materials will be inspected by research assistants before each session. Recruitment will adhere strictly to inclusion/exclusion criteria, and interventions will be delivered at standardized times. Therapists will receive supervision from senior clinicians every 2 weeks. Random recordings of low- and high-functioning group sessions will be reviewed by senior therapists to ensure treatment fidelity. After each session, implementers will complete intervention records documenting duration, content, subjective feedback, and adverse events. Patients considering withdrawal will be counseled on the study’s significance to enhance retention.

During data collection, participants able to complete scales independently will do so; others will respond to researcher-administered questions. Questionnaires will be reviewed on-site for completeness; missing items (>5), consecutive identical responses (≥5), or contradictory answers will result in exclusion as invalid.

Regarding the concealment of group allocation, the personnel responsible for determining the groups shall not participate in the subject enrollment process. The randomization schedule shall be prepared in triplicate and stored in light-opaque envelopes, which will be kept separately by the principal investigator, the study sponsor, and the biostatistician. Any damage to the seal of an envelope must be documented at the time of unblinding. For the handling of unblinding, it shall be performed in the presence of all personnel holding the randomization schedules when the trial is completed or when necessary, with the envelopes opened face-to-face to ensure impartiality. If any abnormality is found on the envelope seal, the reason shall be specified in the report.

Upon completion of follow-up for 50% of participants, an interim analysis of the primary outcome measure will be conducted. This interim analysis will be performed by an independent statistician who is unaware of the specific groupings. The results of the interim analysis will be reported to the study steering committee, which will determine whether to continue the trial and report to the Ethics Committee of Henan Medical University.

Emergency protocols for psychological crises and emotional fluctuations will be in place. Psychiatrists will be on standby throughout the study to ensure patient safety.

### 2.14 Patient and public involvement

No patients or members of the public were involved in the design or implementation of this study. Research objectives, methods, and outcome measures were defined by the research team.

This study has received ethical approval from the Ethics Committee of the Second Affiliated Hospital of Henan Medical University (NO. XXEFYLL-(Research)-2025–131).

## 3 Discussion

Stepwise Yalom group therapy, developed from Yalom’s group therapy model, represents a practical adaptation for Chinese female patients with depressive disorder. As the first study in China to implement a full-course intervention for female inpatients with depressive disorder, it aims to provide evidence for optimizing psychological treatment during hospitalization.

This preliminary exploratory study plans to recruit 90 participants. However, its small sample size limits generalizability across regions, age groups, educational levels, and disease severities—particularly in rural and underserved areas with limited healthcare access. The single-center design further restricts the external validity of results. Additionally, self-reported data may be subject to inherent biases. While a 6-month follow-up is planned, the long-term sustainability of intervention effects remains uncertain.
