# Supplementary material for: A prospective, single-center, quasi-experimental study protocol for evaluating the efficacy of stepwise Yalom group therapy in reducing depressive symptoms and interpersonal problems in Chinese female patients with depressive disorders
Source: Front Psychol. 2026 May 7;17:1831835. doi: 10.3389/fpsyg.2026.1831835 (PMC13190584; doi:10.3389/fpsyg.2026.1831835)
Supplement: Supplementary file 1 [file Data_Sheet_1.pdf]

Intervention content of low-functioning group therapy

(Translated from the Chinese version)

Overall Goal: To provide supportive and structured group experiences for hospitalized patients with low-functioning depression, in order to reduce their sense of isolation and help them acquire basic social skills.

Group setup：
Number of Participants: 4 to 8 people
Leader: 1 therapist
Frequency: 5 times per week, for a total of 1 week
Duration: 30 to 35 minutes each time
Environment: An independent, quiet, undisturbed, and safe treatment room

Note: The five activities can be done in rotation. Participants can join the group in any of the sessions.

Process	Activity Content	Goal	Guidelines and Notes for Therapists
Introduction and Preparation (3 minutes)	Warm but gentle welcome. Emphasize that the group is "a place for rest and support", without pressure, where one can just listen and not speak. Clearly define the basic rules: confidentiality, no criticism, and respecting each person's pace.	Establish a safe and inclusive group atmosphere, reduce anxiety and defensive mentality; clarify group rules to ensure that members feel accepted; welcome new members and promote integration.	The tone is of utmost importance. It should be calm, inclusive and full of hope. At the beginning of each session, new members should be welcomed and the group rules should be briefly introduced.
Warm-up activities (7 to 10 minutes)	Simple ball-throwing game. The therapist needs to demonstrate: "I'm XXX. I'm feeling... / I am... kind of person." Members take turns to catch the ball and upon catching it, they need to say a sentence introducing themselves.	Promote members' mutual acquaintance, reduce the sense of strangeness; encourage simple interactions to complete the "interaction" behavior; reduce social pressure and increase participation.	Significantly reduce the requirements. Just saying the name is sufficient. The warm-up activities for each meeting should include a name-greeting segment to help new members integrate.
Structural discussion (10 to 15 minutes)	Self-disclosure:<br>Draw simple sketches / write something. Members answer questions on paper: One of the most important people in my life is? One thing I really enjoy doing is? Something related to me that others would be surprised to know about?<br>After filling in, members work in pairs, exchange the paper strips to read. If there is anything they don't understand, they can ask the other person to provide additional information. Each pair of members will have a conversation for 3 to 5 minutes before returning to the group. They will take turns sharing the answers of the other person and tell the other members what they have learned from these answers.	Encourage members to expose themselves in a safe environment, starting with their interests, in order to reduce the pressure of exposure; enhance members' interaction and mutual understanding.	The therapist guides the members to disclose their interests first instead of their shortcomings, reducing the pressure of exposure and establishing a secure starting point. The number of things each member exposes themselves should be strictly limited. Self-exposure should be integrated with the focus group's theme of "enhancing member interaction", allowing unfamiliar pairs to work together. If the members have poor comprehension, they can be grouped with the therapist.
Review and Sharing (3 to 5 minutes)	Therapist's summary. Invite members (non-mandatory) to use one word to describe their feelings after the end of the group (such as "safe", "strange", "tired", "happy", etc.).	Consolidate the positive experiences of this meeting; address the feelings of the members, especially the negative ones, so that they are heard and normalized; enhance the cohesion of the group.	Accept all the feelings of the patients, especially the negative ones, and allow them to be heard and normalized.

Process	Activity Content	Goal	Guidelines and Notes for Therapists
Introduction and Preparation (3 minutes)	Warm but gentle welcome. Emphasize that the group is "a place for rest and support", without pressure, where one can just listen and not speak. Clearly define the basic rules: confidentiality, no criticism, and respecting each person's pace.	Establish a safe and inclusive group atmosphere, reduce anxiety and defensive mentality; clarify group rules to ensure that members feel accepted; welcome new members and promote integration.	The tone is of utmost importance. It should be calm, inclusive and full of hope. At the beginning of each session, new members should be welcomed and the group rules should be briefly introduced.
Warm-up activities (7 to 10 minutes)	Activity 1: Name and Greeting: Members stand in a circle and say "I'm XXX" in turn. The group responds with "Hello, XXX".<br>Activity 2: Body Stretching Exercises: The therapist leads the members in simple body stretches.	Promote members' familiarity with each other and reduce tension through physical activities.	Choose the appropriate exercise based on the needs of the members. For example, if some patients complain of discomfort in their shoulders and neck, then they can choose to do exercises for the shoulders and neck.<br>The warm-up activities for each meeting should include a name-greeting segment to help new members integrate.
Structural discussion (10 to 15 minutes)	Empathy:<br>Activity 1: Name Paper Strip Interaction. Each member writes their name on a piece of paper, puts it in a box, and then all members simultaneously draw a name from the box (one at a time, and the drawn papers are not put back until all are drawn). Members take turns to explain to everyone their feelings towards the member whose paper they drew.<br>Activity 2: Human Outline Map Interaction. Give each member a piece of paper with a human outline on it. Members mark the parts they are satisfied with on the map, write their names, and pass it to the person next to them. The member who receives the paper marks the parts they like on the person whose name is on the paper. After passing around once, the paper returns to the original member. Check how many marked parts there are. Each member takes turns expressing their thoughts and feelings about the marked parts.	Members develop the ability to pay attention to and understand others' feelings by drawing names and sharing their thoughts. Through the use of body outline diagrams for annotation and sharing, members are guided to focus on themselves and others, and diverse expressions are used to promote mutual understanding, gradually enhancing their empathy.	Guide members to avoid negative and critical emotions towards others, encourage supportive interactions, and focus on the positive aspects of others.
Review and	Therapist's summary. Invite members (non-mandatory) to use	Consolidate the positive experiences	Accept all the feelings of the patients,

Sharing (3 to 5 minutes)	one word to describe their feelings after the end of the group (such as "safe", "strange", "tired", "happy", etc.).	of this meeting; address the feelings of the members, especially the negative ones, so that they are heard and normalized; enhance the cohesion of the group.	especially the negative ones, and allow them to be heard and normalized.

Process	Activity Content	Goal	Guidelines and Notes for Therapists
Introduction and Preparation (3 minutes)	Warm but gentle welcome. Emphasize that the group is "a place for rest and support", without pressure, where one can just listen and not speak. Clearly define the basic rules: confidentiality, no criticism, and respecting each person's pace.	Establish a safe and inclusive group atmosphere, reduce anxiety and defensive mentality; clarify group rules to ensure that members feel accepted; welcome new members and promote integration.	The tone is of utmost importance. It should be calm, inclusive and full of hope. At the beginning of each session, new members should be welcomed and the group rules should be briefly introduced.
Warm-up activities (7 to 10 minutes)	Activity 1: Name Chain: Members stand in a circle and take turns saying "I am XXX". Then, "I am the XXX next to me", "I am the XXX next to the XXX next to me", and so on.<br>Activity 2: Find the Differences: The therapist guides the group members to observe each other carefully. Then, a "lucky" member is asked to leave the group for a few minutes. The remaining members are required to make slight changes to their appearance (such as rolling up their sleeves, taking off their glasses, picking up a towel, etc.). When the member returns, they need to point out the changes made by the other members.	Enhance members' attention to each other, and promote observation and interaction.	Members who are willing to take the initiative can be given priority to be selected as the "lucky members".
Structural discussion (10 to 15 minutes)	The interaction at this moment and this place:<br>Activity One: Pair up and find similarities and differences. Two people form a pair and identify the two places where they are the same and the two places where they are different. Each pair takes turns to share the discussion content with the group.<br>Activity Two: Fill-in-the-blank sentences (focusing on the group's daily life). Members complete short sentences on paper: In this group, the reason why I feel envious of someone isXXX. After completion, members take turns to read out their answers and give a brief introduction.	Help members focus on the immediate interactions and feelings within the group, and enhance mutual understanding.	The answers are not right or wrong; they merely express feelings. They focus on the daily life of the group and enhance mutual understanding and inner expression among members. Immediate discussions and feedback are conducted around the current conversations, emotional reactions, interaction patterns, etc. that occur within the group.<br>The therapist needs to guide the group members to explore various communication and interaction processes that take place within the group, helping the members understand what has happened within the

			group and "what is happening at this moment and in this place".
Review and Sharing (3 to 5 minutes)	Therapist's summary. Invite members (non-mandatory) to use one word to describe their feelings after the end of the group (such as "safe", "strange", "tired", "happy", etc.).	Consolidate the positive experiences of this meeting; address the feelings of the members, especially the negative ones, so that they are heard and normalized; enhance the cohesion of the group.	Accept all the feelings of the patients, especially the negative ones, and allow them to be heard and normalized.

Process	Activity Content	Goal	Guidelines and Notes for Therapists
Introduction and Preparation (3 minutes)	Warm but gentle welcome. Emphasize that the group is "a place for rest and support", without pressure, where one can just listen and not speak. Clearly define the basic rules: confidentiality, no criticism, and respecting each person's pace.	Establish a safe and inclusive group atmosphere, reduce anxiety and defensive mentality; clarify group rules to ensure that members feel accepted; welcome new members and promote integration.	The tone is of utmost importance. It should be calm, inclusive and full of hope. At the beginning of each session, new members should be welcomed and the group rules should be briefly introduced.
Warm-up activities (7 to 10 minutes)	Activity 1: Name and Greeting: Members stand in a circle and say "I'm XXX" in turn. The group responds with "Hello, XXX".<br>Activity 2: Emotional Weather Report: "If your mood were like a weather condition, would it be sunny, cloudy, rainy, light rain or heavy rain?" Have members take turns sharing.	Encourage members to express their current emotions.	Records must be kept.
Structural discussion (10 to 15 minutes)	Explanatory discussion:<br>The therapist briefly explained: "Emotions are like the weather and can change. We can prepare an 'umbrella' for ourselves." She encouraged the members to brainstorm "What very simple things can you do to take care of yourself when you feel down/tense/sad...?" (For example, "Take a hot shower", "Listen to a song", "Hug a pillow"). She guided the members to write the list on paper at any time.	Summarize simple strategies for emotional regulation and enhance one's ability to take care of oneself.	Through guided discussions or brief explanations, encourage members to actively participate and think of more effective solutions. The list can be kept by the patients themselves.
Review and Sharing (3 to 5 minutes)	Therapist's summary. Invite members (non-mandatory) to use one word to describe their feelings after the end of the group (such as "safe", "strange", "tired", "happy", etc.).	Consolidate the positive experiences of this meeting; address the feelings of the members, especially the negative ones, so that they are heard and normalized; enhance the cohesion of the group.	Accept all the feelings of the patients, especially the negative ones, and allow them to be heard and normalized.

Process	Activity Content	Goal	Guidelines and Notes for Therapists
Introduction and Preparation (3 minutes)	Warm but gentle welcome. Emphasize that the group is "a place for rest and support", without pressure, where one can just listen and not speak. Clearly define the basic rules: confidentiality, no criticism, and respecting each person's pace.	Establish a safe and inclusive group atmosphere, reduce anxiety and defensive mentality; clarify group rules to ensure that members feel accepted; welcome new members and promote integration.	The tone is of utmost importance. It should be calm, inclusive and full of hope. At the beginning of each session, new members should be welcomed and the group rules should be briefly introduced.
Warm-up activities (7 to 10 minutes)	Activity 1: Name and Greeting: Members stand in a circle and say "I am XXX" in turn. The group responds with "Hello, XXX".<br>Activity 2: "I Hope..." Relay: Members take turns to complete the sentence: "For myself in the future, I have a little hope: " (e.g. "I hope to have a good sleep", "I hope to be able to laugh one day").	Encourage a positive outlook on the future.	Focus on "hope" rather than "goals", and reduce the stress on members.
Structural discussion (10 to 15 minutes)	Self-Change:<br>Sentence Completion. Members should first complete two short sentences on paper: 1. The two changes I want to make are? 2. My thoughts on how to achieve these two changes are? After completion, members work in pairs to read each other's answers, understand the ways their partners have implemented the changes, and give suggestions to promote the changes. After the discussion, members stop the discussion on their own, each member takes turns introducing their partner to the other members of the group, describing the methods their partner proposed to achieve the changes, and giving suggestions to promote their partner's change. All members add their suggestions.	Encourage members to reflect on personal changes and develop plans with the support of the group.	Pay attention to guiding the members to express their opinions. If the patient says "I don't want to change", it is not appropriate to force the members to do so.
Review and Sharing (3 to 5 minutes)	Therapist's summary. Invite members (non-mandatory) to use one word to describe their feelings after the end of the group (such as "safe", "strange", "tired", "happy", etc.).	Consolidate the positive experiences of this meeting; address the feelings of the members, especially the negative ones, so that they are heard	Accept all the feelings of the patients, especially the negative ones, and allow them to be heard and normalized.

		and normalized; enhance the cohesion of the group.	
