# Supplementary material for: A prospective, single-center, quasi-experimental study protocol for evaluating the efficacy of stepwise Yalom group therapy in reducing depressive symptoms and interpersonal problems in Chinese female patients with depressive disorders
Source: Front Psychol. 2026 May 7;17:1831835. doi: 10.3389/fpsyg.2026.1831835 (PMC13190584; doi:10.3389/fpsyg.2026.1831835)
Supplement: Supplementary file 2 [file Data_Sheet_2.pdf]

Intervention content of high-functioning group therapy

(Translated from the Chinese version)

Overall Goal: To help patients with depression disorders identify and correct negative interpersonal patterns, learn new social skills through "in the moment" interactions, alleviate interpersonal distress, thereby reducing depressive symptoms, enhancing social functions, and promoting recovery.

Group Setup:
Number of Participants: 8 to 12
Leader: 1 therapist
Frequency: 3 times per week for a total of 4 weeks
Duration: 60 to 75 minutes each time
Environment: An independent, quiet, undisturbed, and safe treatment room

Process	Activity Content	Goal	Guidelines and Notes for Therapists
Introduction and Preparation (3 to 5 minutes)	The therapist introduces herself and informs the group members of the basic process and goals of this group therapy session, as well as the duration, objectives, procedures and confidentiality principles of the therapy.	Establish a group framework, clarify expectations and rules, and prepare for the treatment.	If there are new members joining, detailed introductions need to be given.
Introduce personal topics (20 to 30 minutes)	The therapist guides the members to take turns speaking and raises each issue one by one (the issues should be realistic, solvable through communication with the members, and related to interpersonal relationships). The therapist needs to assist the members in formulating the issues, transforming them into interpersonal problems and integrating them into the current therapeutic context.	Encourage members to identify and express their personal interpersonal issues, providing materials for interactive discussions.	Members may have difficulty accurately understanding the therapist's intention and meaning when the therapist asks them to raise issues. The therapist must explain this concisely and clearly. If necessary, the therapist should provide the patient with appropriate case examples of issues and patiently help the members construct their own issues. When combining issues, it is necessary to balance "commonality" and "individuality", be vigilant against "over-combination", and promptly split the discussion when members emphasize "my situation is different", to avoid ignoring unique experiences. Pay attention to "silent members". After combining issues, proactively ask "Is there anything you particularly want to add to this issue?" to leave room for personalized expression.
Discuss personal matters (20 to 35 minutes)	The therapist guides the members to take turns speaking, and the group organizes itself to solve problems through discussion.	Analyze interpersonal issues through group interaction and learn social skills.	The general principles of the discussion topics: Enable more members to obtain the maximum benefits, handle multiple topics simultaneously, actively handle the topics, handle them directly or indirectly, and go beyond the topics.
Therapist's summary (10 minutes)	The therapist reviews the entire process of the session, the atmosphere of the group, the level of interaction, the conflicts among members, etc. They review the decisions made by the members during the session, reflect on their own leadership style (whether any omissions occurred, whether there were better ways to handle things, etc.). They focus on the performance of each member (the type of issues, the level of initiative or participation, the progress in handling the issues, etc.), and briefly analyze and evaluate the satisfaction of each	Integrate the conversation content, provide feedback, and enhance learning.	Mainly provide positive, supportive and constructive suggestions, so that members can feel valued and encouraged.

	patient.		
Feedback from group members (10 minutes)	Members discussed the therapist's analysis and summary, presented their own analysis and summary for the group activities, or continued to discuss the unfinished matters from the previous group activities.	Encourage members to reflect and express themselves, and enhance their sense of participation.	Technique: Structured guidance. Use the template of "feeling, suggestion, gain, outlook" to provide directions for team members, avoiding the awkward situation of "having nothing to say".
Note: When the patient raises personal issues, the therapist should consciously guide and focus the topic on interpersonal problems. When multiple issues are combined, the therapist should have the ability to go beyond the surface content of the issues and extract the underlying common interpersonal problems. For example, situations like "Others keep making me do things I don't like" and "I don't want to go out to play, but she keeps calling me to go out" may be combined into the interpersonal issue of "Not knowing how to firmly and politely refuse others"; "Being afraid of offending others when speaking" and "Feeling very uncomfortable when no one responds to my opinions" can be refined into "Overly caring about others' evaluations and difficulty in expressing one's true self".			
