# Supplementary material for: A prospective, single-center, quasi-experimental study protocol for evaluating the efficacy of stepwise Yalom group therapy in reducing depressive symptoms and interpersonal problems in Chinese female patients with depressive disorders
Source: Front Psychol. 2026 May 7;17:1831835. doi: 10.3389/fpsyg.2026.1831835 (PMC13190584; doi:10.3389/fpsyg.2026.1831835)
Supplement: Supplementary file 3 [file Data_Sheet_3.pdf]

Stepwise Yalom Group Therapy Treatment Fidelity Checklist

(Translated from the Chinese version)

I. Instructions for Use

Purpose of Evaluation: To assess whether the group therapy session was conducted in accordance with the pre-set operation manual, and to record the therapist's consistency in execution, stage appropriateness, and any deviations from the plan.

Scoring Method: Score each item as follows:

0= Not implemented / Clearly does not conform

1= Partially implemented / Quality is average

2= Fully implemented / Conforms to manual requirements

NA= Not Applicable

II. Basic Information

Evaluation Date:

Evaluation object: ☐ Low-functioning group /☐ High-functioning group

Valuer:

The evaluated therapist:

Planning duration:

Actual duration:

III. General Items

**Item**	**Score (0/1/2/NA)**	**Remark**
1. Carry out the treatment in an independent, quiet, undisturbed and safe room.		
2. The therapist completes the opening introduction and explains the current process/aim/duration.		
3. The therapist emphasized the basic rules of confidentiality, respect and absence of criticism.		
4. A necessary introduction and integration guidance were provided to the new members.		
5. Basically, we are proceeding in accordance with the procedures specified in the manual.		
6. The therapist maintained a supportive and non-judgmental atmosphere.		
7. The therapist responds appropriately to silence, withdrawal, mood swings or difficulties in participation.		
8. The therapist encouraged the members to participate and did not use coercive methods.		
9. The therapist concluded by providing a summary/share.		
10. Records were made of any obvious deviations from the plan, unexpected incidents, or adverse reactions from the members.		
Total		

IV. Evaluation of Low-Functioning Groups

Item	Score (0/1/2/NA)	Remark
**Duration and Structure**		
1. The total duration should be kept within 30 to 35 minutes.		
2. Completed the opening preparations (approximately 3 minutes)		
3. The warm-up phase has been completed (lasting approximately 7 to 10 minutes)		
4. The structured discussion session was completed (lasting approximately 10-15 minutes)		
5. The review and sharing session has been completed (lasting approximately 3-5 minutes)		
**Core principles of low-functioning groups**		
6. The opening remarks are gentle, calm, inclusive, and convey a sense of hope.		
7. Clearly convey the message that "One can simply listen without speaking, and there is no pressure."		
8. The requirements for participation have been significantly reduced, allowing members to join the activity in the least demanding way.		
9. The therapist avoids criticism, negation or over-explanation.		
10. The therapist assists the members in carrying out the action of "entering the interaction"		
11. The therapist adjusts the difficulty level of the activities flexibly according to the members' conditions.		
12. Accept, name and normalize negative emotions		
13. The therapist did not force the members to undergo self-disclosure or make any "changes"		
Total		

V. Evaluation Items for High-Functioning Groups

Item	Score (0/1/2/NA)	Remark
**Duration and Structure**		
1. The total duration should be controlled within 60 to 75 minutes.		
2. Completed the opening preparations (approximately 3 minutes)		
3. The introduction of the personal topic segment was completed (lasting approximately 20-30 minutes)		
4. The individual topic discussion session was completed (lasting approximately 20-35 minutes)		
5. The therapist's summary session was completed (lasting approximately 10 minutes)		
6. The member feedback session has been completed (approximately 10 minutes).		
**Core principles of high-functioning groups**		
7. The therapist assists the members in identifying "real, discussable, and interpersonal-related" personal issues.		
8. The therapist appropriately transforms/abstracts the individual issues into interpersonal problems.		
9. When integrating the topics, consider both commonalities and individual uniqueness, and avoid "excessive merging"		
10. The therapist noticed the silent member and provided an appropriate opportunity for them to speak.		
11. The therapist facilitates the present-moment process, that is, focuses on the ongoing interactions taking place within the group at this moment.		
12. The therapist promptly addressed conflicts, differences, or avoidance behaviors.		
13. The feedback provided by the therapist is mainly composed of positive, supportive and constructive suggestions.		
Total		
